# Revisiting the outstanding questions in cancer nanomedicine with a future outlook

**DOI:** 10.1039/d1na00810b

**Published:** 2021-12-22

**Authors:** M. S. Sudheesh, K. Pavithran, Sabitha M

**Affiliations:** Dept. of Pharmaceutics, Amrita School of Pharmacy Amrita Health Science Campus, Amrita Vishwa Vidyapeetham, Ponekkara Kochi – 682041 India sabitham@aims.amrita.edu mssudheesh@aims.amrita.edu +91-9669372019; Department of Medical Oncology, Amrita Institute of Medial Sciences and Research Centre Amrita Health Science Campus, Amrita Vishwa Vidyapeetham, Ponekkara Kochi – 682041 India

## Abstract

The field of cancer nanomedicine has been fueled by the expectation of mitigating the inefficiencies and life-threatening side effects of conventional chemotherapy. Nanomedicine proposes to utilize the unique nanoscale properties of nanoparticles to address the most pressing questions in cancer treatment and diagnosis. The approval of nano-based products in the 1990s inspired scientific explorations in this direction. However, despite significant progress in the understanding of nanoscale properties, there are only very few success stories in terms of substantial increase in clinical efficacy and overall patient survival. All existing paradigms such as the concept of enhanced permeability and retention (EPR), the stealth effect and immunocompatibility of nanomedicine have been questioned in recent times. In this review we critically examine impediments posed by biological factors to the clinical success of nanomedicine. We put forth current observations on critical outstanding questions in nanomedicine. We also provide the promising side of cancer nanomedicine as we move forward in nanomedicine research. This would provide a future direction for research in nanomedicine and inspire ongoing investigations.

## Abstract 2

EPR, enhanced permeability and retention effect; ABC, accelerated blood clearance; TME, tumour microenvironment.

## Graphical abstract

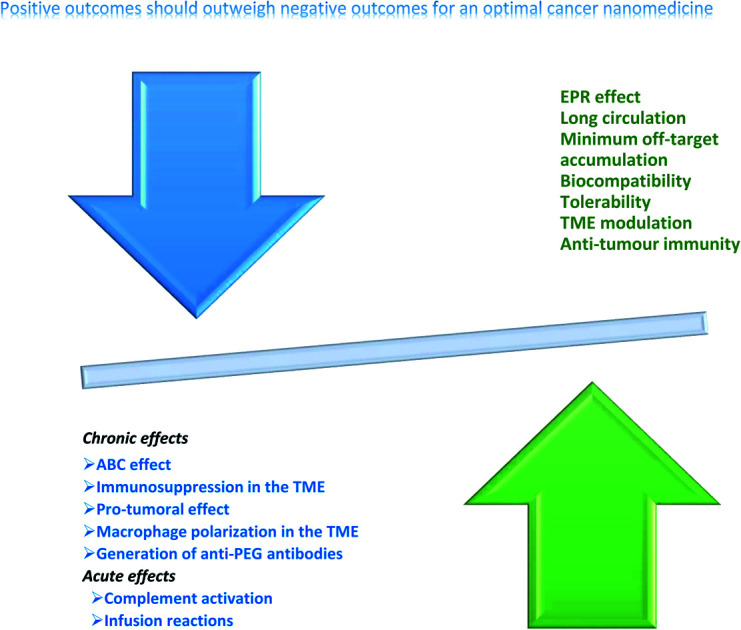

## 1. A brief historical background

The history of the evolution of drug delivery systems (DDSs) is a story of triumphs and failures as we moved from simple to complex drug delivery approaches. DDSs have been classified into different generations (Table 1).^1^ Delivery systems of the first generation (1G) were the most successful in terms of the number of commercial products. The success of the 1G DDSs is attributed to the temporal control of drug release by rate-controlled processes. The control on drug release was based on the understanding of pharmaceutical factors that influence drug release such as solubility, diffusion, dissolution and osmosis. Precise engineering of delivery systems and mathematical models of drug release played an important role in the success of these systems. One of the major shortcomings of 1G-DDSs was the lack of control on the spatial distribution of the drug in the body after being released from the DDS. In 2G-DDSs the same technology was adapted for slow release of protein-based pharmaceuticals in the form of implants and depot formulations. But these formulations could not succeed as, unlike small molecules, solid-state stabilization of biomolecules like proteins in their native state is a challenging task. Moreover, immunogenicity due to misfolding of therapeutic proteins during formulation development is a major drawback of these formulations. The formulation performance was also influenced by the interaction of proteins and cellular components with the implants. Loss of functionality and immunogenicity due to misfolding/aggregation of the native protein was a major impediment in the success of these types of systems. Further, preclinical animal models were not able to predict the immunogenicity of the proteins released from polymeric matrix formulations that were later found to be highly immunogenic in human trials.^2^

**Table 1:** Characteristics of different generations of DDSs

Gen	Approaches	Mechanism	Inspired by	Shortcomings	Products
1G	Rate controlled delivery, slow release	Solubility, diffusion and dissolution-controlled mechanism	Mathematical models of drug release and polymer chemistry	No spatial control	Oral and transdermal systems
2G	Rate controlled delivery of proteins, zero order release spatial controlled approaches	Polymer controlled release from implants, nanoscale properties such as the size, charge *etc.*	Solid-state protein stabilization EPR effect, and stealth effect	Immunogenicity of the released protein molecules, and lack of control over distribution	Slow-release implants and targeted nanoparticles
3G	Approaches to improve NP targeting, modulated release by implants and stimuli sensitive polymers	>6 months protein release, non-invasive delivery, cancer targeting	EPR effect, stealth effect, biodegradable polymers, and gene delivery vehicles	Poor clinical translation due to poor efficacy and lack of IVIVC^a^	Depot formulation, liposomal formulation and NPs

a IVIVC: *in vitro in vivo* correlation.

The market withdrawal of Nutropin Depot™ and Exubera™ is an example of the problems associated with protein formulations. However, the initial success of 1G-DDSs fueled the future generations of delivery vehicles *i.e.*, 2G & 3G-DDSs. One of the important inclusions is the targeted nanomedicines which promise a high degree of spatial targeting by delivering the payload directly to the diseased cells and tissues. Conceptually, this strategy was thought to be highly efficient as it would reduce the off-target effects and show optimal activity at a fraction of the dose required by conventional systems. In 1G DDSs, engineering of the device for temporal control was the focus of the drug delivery approach, and no attempts for spatial control were undertaken. In the 2G DDS attempts were made to control the temporal and spatial distribution of drugs, which were further optimized for different applications such as gene delivery and self-regulated protein delivery in the 3G DDS (Table 1). However, a poor *in vitro in vivo* correlation (IVIVC) was a major drawback of 3G DDSs. The human body has a complex set of multi-tiered barriers to protect it from unwarranted entry of foreign invaders which is a result of thousands of years of the evolutionary process. For spatial control, dealing with the complexity of human biological barriers is the most limiting factor. Apart from these barriers, the pathological state of diseased tissues *e.g.* tumour tissues adds an additional layer of complexity in the form of a hostile tumour microenvironment (TME).^3^

**Fig. 1:**
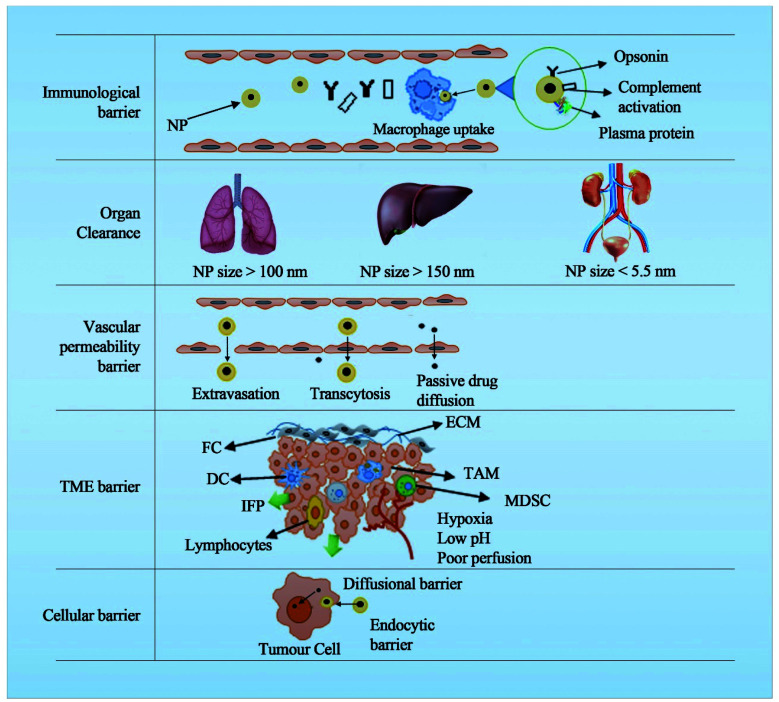
Different levels of anatomical and physiological barriers in clinical translation of cancer nanomedicine. (TAMs, tumour associated macrophages; NPs nanoparticles; DCs, dendritic cells; FC fibroblast cell; IFP interstitial fluid pressure; MDSC, myeloid-derived suppressor cells; TME tumour microenvironment; ECM extracellular matrix).

The presence of multiple barriers requires complex engineering to deal with the physiological and anatomical aspects of these biological barriers. For *e.g.* NPs for cancer should cross several barriers such as the immunological barrier, the vascular barrier, the tumour extracellular barrier, the tumour microenvironment and the cellular barrier (Fig. 1). The nanoscale properties of the NPs are expected to help safely navigate through these barriers to deliver the payload at the tumour site by immune evasion, extravasation and endocytic mechanisms. As the generation of DDSs increased, the rate of clinical translation reduced progressively mainly because of a poor understanding of the complex biological barriers.

The field of nanomedicine represents the convergence of the complexity of the biological field and the precise quantitative approaches used in the physical sciences. A lack of communication between disciplines has been a major challenge. The tendency to oversimplify and generalize the complexities and redundancy of biological systems by physical scientists and the lack of understanding of physical properties at the nanoscale among biologists have led to misperceptions and inappropriate conclusions.^4^ An overemphasis on the engineering aspect of NPs and an under-appreciation for the complex biological and immunological consequences of these delivery approaches are partly responsible for the low success rate of these delivery systems. The trends in publications also exemplify this as a large part of the work on cancer nanomedicine is published in materials science, chemical engineering and related fields and relatively a low percentage of articles (17.3%) have been published in medical and biological science-related journals (based on the data from Scopus by Salvioni *et al.*, 2019).^5^

Opinions are divided for^6^ and against^7,8^ the efficiency and success of nanomedicine in cancer therapeutics. Some of the recent discussions on the future of the field have led to a lot of skepticism with diametrically opposite views and opinions.^9,10^ Existing paradigms in nanomedicine are debated, discussed and questioned. New results in the field are counterintuitive to the existing dogmas including the underlying assumption that solid tumours could be targeted by the enhanced permeability and retention effect (EPR effect) and the long circulatory “stealth effect” of PEGylated NPs.^9,11,12^ These observations have raised even more questions on the existing fundamentals of nano-drug delivery. However, every new question also brings new opportunities and dimensions for growth. Nanomedicine-based immunotherapy against cancer is an example of an alternate strategy in cancer nanomedicine.^13^ The present review is an attempt to analyse the questions that have been raised regarding the fundamental principles of nanomedicine. We also discuss the implications of the new developments in the field on approaches for the future generations of DDS.

## 2. Passive targeting of cancer nanomedicine

### 2.1. Status of the ‘EPR effect’ and clinical relevance

The initial success in cancer nanomedicine was fueled by the concept of enhanced permeability and retention (EPR effect) observed in pre-clinical models.^14^ EPR has been questioned and discussed critically^15^ and this section will only highlight briefly some latest observations. A leaky vasculature due to neovascularization, wider fenestrated endothelium, poor perfusion and poor lymphatic drainage is thought to be the driver for enhanced extravasation and sequestration of NPs in the tumour architecture.^16,17^ EPR has dominated the literature on cancer nanomedicine as the primary reason for the efficacy of NPs. However, blatant misuse of this concept to claim therapeutic superiority has been at the epicenter of the current debate surrounding the efficiency of nanomedicine.^7,18^ The main criticism against the EPR effect is that the success of pre-clinical effects could not be translated into clinically significant efficacy. The lack of EPR effect has been attributed to a large inter- and intra-individual heterogeneity in the tumour vasculature which is highly variable at different stages of tumorogenesis and depends on the tumour type.^19^ The difference in tumor vascular permeability which is related to poor diffusion and penetration of NPs between a xenograft model and a clinical tumor has been claimed as the reason for poor clinical translation of NPs.^16,20,21^ The vascular structure should resemble the clinical tumour for developing predictive preclinical models.^20^ The vascular pore size between endothelial cells of the xenograft model may vary from hundred nanometers to micrometer size which can influence vascular permeability and the EPR effect.^16^ The vascular permeability also varies with the site of tumour implantation.^21^ Kataoka *et al.* gave a radically different view of the mechanism of tumour accumulation.^22^ They used intravital confocal laser scanning microscopy to observe a dynamic phenomenon called the vascular burst characterized by time-limited eruption of blood vessels which results in an outward flow of fluid to the interstitial tissue space. These transient pores formed in the blood vessels known as the ‘dynamic vents’ change the distribution pattern of NPs. A limited accumulation of 30 nm tracer NPs as compared to 70 nm particles was observed due to the dynamic vents formed in the blood vessels.

The inter endothelial gap has been used as a rationale for the EPR effect.^16^ However, recently it has been reported that these gaps are not responsible for the entry of NPs into the tumour tissue. Instead, 97% of the NPs enter tumour cells by an active transcytosis mechanism through endothelial cells.^12^ This is disruptive in the sense that it contradicts the currently held notion of the EPR effect according to which, NPs extravasate by a passive transport mechanism. Further, a high percentage of the systemically administered (88.2–99.9%) active targeted NPs are retained in the acellular region in the tumour microenvironment (TME).^23^ A perspective article by Wilhelm *et al.* has been at the centre of controversy and highly debated recently. It is based on a meta-analysis of the literature on cancer targeting according to which only 0.7% (median) of the administered NP dose is delivered to the tumour.^11^ This has been highly contested by another recent article refuting the claim by Wilhelm *et al.*^24^ The data were reanalysed using classic PK metrics and 100 times higher tumour delivery by NPs was reported using (AUC_tumor_/AUC_blood_) as compared to % injected dose (%ID) used by Wilhelm *et al.*^11^ The only approved nanoformulation that shows prolonged patient survival compared to the current standard of treatment is a recently approved liposomal formulation of a combination of two cytotoxic drugs (daunorubicin and cytarabine, Vyxeos). Vyxeos is indicated for acute myeloid leukemia wherein EPR is not expected to be the driver of efficacy,^25^ despite neovascularization in the bone marrow.^26,27^ These observations challenge our current rationale for the development of passive targeted cancer nanomedicine by the ubiquitous gateway of the EPR effect.

### 2.2. The ‘stealth effect’ and tumor-targeting

PEGylation is the most widely used strategy to develop long circulatory NPs which is believed to enhance passive targeting. PEG grafting on the surface of nanocarriers has been the gold standard in surface passivation of NPs and is critical for long circulation and the stealth effect. However, similar to the EPR effect, the ‘stealth effect’ which is one of the guiding principles in cancer targeting has been actively scrutinised for its proposed claims. PEG was thought to create a non-fouling surface by preventing adsorption of plasma proteins especially opsonins which drive the process of opsonization.^28^ Recently however this paradigm has been challenged and the current view is that protein adsorption, in fact, takes place on PEG carriers and it is practically impossible to create a completely non-fouling surface. The stealth effect is not a polymer effect alone, but it involves a secondary effect due to the adsorption of biocompatible macromolecules from plasma. Schöttler *et al.* (2016) reported that the PEGylated nanocarriers evade MPS uptake by adsorbing specifically the plasma protein called clusterin (also known as apolipoprotein J) which belongs to a class of molecule called dysopsonins.^29^ Dysopsonins are the so-called “don't eat me signal” and their adsorption on the NP surface prevents the recognition and uptake of the MPS by the cells. Other examples of dysopsonin are CD 47, histidine rich glycoprotein (HRG), Apo A4, Apo C3 and human serum albumin.^30,31^ Clusterin also suppresses the cell uptake of non-pegylated NPs like silver and silica nanoparticles.^32^ Clusterin with a molecular weight of 80 kDa acts as a molecular chaperone and binds to hydrophobic domains of unfolded proteins and prevents protein aggregation.^33^ It has been speculated that PEG molecules on the NP surface are mistaken for aggregated proteins resulting in the binding of clusterin which acts as a molecular chaperone by refolding proteins to their native conformation.^29^ Clusterin has a strong dysopsonin property regardless of the surface on which it is adsorbed. In contrast, the adsorption of opsonins such as immunoglobulins, complement proteins, fibrinogens *etc.* on NPs is responsible for rapid clearance of NPs by opsonization. Opsonization can also happen without protein adsorption as demonstrated in protein depleted media which shows the existence of alternate mechanisms for cell uptake.^29^

Questions have been raised against the use of stealth NPs for tumour targeting.^34^ Long circulating PEGylated liposomes neither extravasate substantially to the tumour tissue nor they are cleared by the MPS. It has been observed that long circulation is associated with skin deposition of PEGylated liposomal doxorubicin (PLD), causing incidence of dermal toxicity, which is not observed in non-PEGylated liposomes.^35,36^ The long circulatory behaviour promotes the kinetically slow process of extravasation into the skin.^37^ A comparison of commercial PEGylated (Doxil™) and non-PEGylated liposomes (Myocet™) shows that changes in pharmacokinetic parameters due to PEGylation does not contribute to efficacy.^38^ Doxil™ and Myocet™ differ in terms of the lipid composition, drug release and circulation half-life. Due to high drug release, only 10% of the drug is retained by Myocet™ after 24 h, whereas even after 2–3 days of circulation 50% of the dose is retained in Doxil™, which in principle should promote EPR and enhance efficacy. In contrast, phase III trials show no difference in efficacy while dermal side effects seen with Doxil™ are practically eliminated with Myocet™.^39,40^ Collectively, these pieces of evidence challenge the currently held notion that long circulation will provide additional time for NPs to extravasate into leaky tumour tissue, thereby contributing to the EPR effect.

PEGylation, on the one hand, increases the circulation time of NPs by evading cell uptake by the MPS and, on the other hand, it prevents the internalization and endocytosis of NPs by the tumour cells resulting in limited therapeutic efficacy. This is called the “PEG dilemma”. A strategy to overcome this problem is to design a cleavable PEG corona in response to environmental stimuli. These stimuli include acidic pH, hypoxia and the presence of certain enzymes in the TME.^41,42^ However, due to inter- and intra-heterogeneity^43,44^ in the TME and the transient nature of the stimuli, these approaches suffer from variability similar to the variability of the EPR effect.

### 2.3. Safety and immunocompatibility of PEGylated carriers

The PEGylation strategy has been used for the past more than two and a half decades in commercial nanomedicine formulations with the introduction of the first PEGylated liposomal doxorubicin (PLD) Doxil™. However lately, it has been observed that PEGylation is associated with mild to very strong immunological reactions which led to the clinical failure of some PEG-conjugated drugs. PEGylation has been linked to the death of some patients during an infusion reaction which led to the market withdrawal of PEGinesatide (a functional analog of erythropoietin).^45^ The failure in the clinical trial of a PEGylated aptamer has been linked to PEGylation and anti-PEG antibodies.^46^ Following these incidences, the FDA has revised the guidelines and has recommended screening of anti-PEG antibodies in patients.

Anti-PEG antibodies are associated with mild to severe life-threatening infusion reactions (IR) and represent a translational hurdle for NP-based products.^47^ The mechanism of these IRs is poorly understood. The binding of anti-PEG IgM antibodies to PEGylated NPs has been reported to cause IRs by inducing a complement reaction and anaphylactoid shock.^48^ Despite several studies, the cause-effect relationship of the physicochemical attributes of NPs with complement activation, cell uptake and cytokine release are still unclear.^47^ Differences in vesicle-mediated complement response have been observed for two different brands of PEGylated liposomes (Doxil™ and Caelyx™) which are perceived to be similar.^49^ The role of platelets as an effector for a complement response is also being suspected.^50,51^ Complement activation-related pseudoallergy (CARPA) syndrome is one of the underlying mechanisms of IR.^52,53^ Pre-existing anti-PEG antibody is regarded as one of the most critical factors for complement activation by PEGylated NPs.^54^ The complement activation has been found to increase linearly with the concentration of anti-PEG antibodies during a second injection with PEG.^55^ Anti-PEG antibodies can induce a complement-dependent disruption of the liposomal membrane resulting in rapid drug release from liposomes. PEG is not able to prevent the binding of complement fragment C5b-9 on liposomes and the release of drug that is thought to happen due to the disruption of the proton and ammonium ion gradient in PLD used for passive drug loading.^56^ Anti-PEG antibodies could be a factor responsible for the variable effect of PEGylated liposomes. A concentration above the cut-off anti-PEG IgG titre required for liposomal membrane disruption has been estimated to be present in 5.5% of the normal population.^56^ A minimal physiologically based pharmacokinetic (PBPK) model predicts that a median concentration of 50 ng ml^−1^ of pre-existing anti-PEG antibody may only result in a 5–15% decrease in AUC relative to patients having no anti-PEG antibody.^57^ A genetic basis for the generation of anti-PEG antibodies has also been identified since only selective patients show the propensity to induce them which increases the risk of IR. A genome-wide association study shows that the immunoglobulin heavy chain is the susceptible locus for an anti-PEG IgM response.^58^

The PEG-liposome–IgM complex is also responsible for a phenomenon called accelerated blood clearance (ABCs) by which NPs are rapidly removed from circulation after a second injection of PLD.^59^ However, it is intriguing that the ABC phenomenon has not been observed in clinical studies, which is well-established in different animal models. Two reasons for this observation are proposed in the literature. First, the immunological tolerance of PEG at a higher dose and second, the cytotoxic effect of PLD on macrophages.^60^ There is evidence to both the claims. It is also reported that there is an inverse correlation between the initial dose of PLD and the extent of ABC.^61^ The administration of a high dose (equivalent to the clinically recommended dose of PLD) results in the abrogation of the ABC phenomenon. This has been attributed to a state of immunological tolerance or clonal anergy of the marginal zone B cells (which trigger the generation of anti-PEG IgM).^62,63^ This occurs at a high dose of PLD due to the lack of appropriate co-stimulatory signals. However, similar effects were not observed with PEGylated polymeric carriers at a higher dose.^64^ The hepatic clearance of the PEGylated liposome shows a sigmoidal relationship with anti-PEG antibodies which is attributed to the capacity-limited uptake by the Kupffer cells.^55^ In fact, the hepatic clearance of NPs and not the anti-PEG antibody-mediated complement activation is the limiting step in ABC.

Contrary to ABC observed in animals, human clinical studies show that the clearance of PLD was progressively reduced from cycle 1 to cycle 3 of chemotherapy.^65,66^ There is a clear dose-dependent reduction of the monocyte count which is attributed to the cytotoxic effect of the drug, doxorubicin. Age and gender-related changes in clearance are also associated with a change in the MPS function.^66^ This also highlights the fact that study in rodent models is of limited value especially when immunological processes are responsible for the pharmacokinetic clearance of the NPs. A memory response mediated by the anti-PEG antibody is thought to be responsible for the rapid clearance of PEG-based therapeutics.^57^ It is critical for physicians who regularly prescribe PEGylated therapeutics to know the implications of anti-PEG antibodies. An interesting survey suggests that only roughly one-quarter of the physicians who prescribed PEGylated therapeutics knew of the presence of PEG in the formulation and that this formulation can generate anti-PEG antibodies and its implications to patient safety.^67^

## 3. Biomolecular corona and tumor-targeted nanomedicine

### 3.1. Biomolecular corona (BC) and the fate of NPs

The high surface free energy of NPs inevitably attracts plasma proteins (including antibodies and complement proteins) present in blood almost instantaneously on their surface which is called the biomolecular corona (BC).^68^ Individual variability in the blood proteome and adaptive immunity can result in different personalized BC. The newly acquired biological identity (personalized BC) shields the original physicochemical properties of NPs, making them vulnerable to variable host–immune reactions. BC can also shield active targeting ligands (present on NP surface) from binding to its intended target/receptor on cells.^69^ Engineering the surface properties of NPs should take into account the events at the nano-bio interface. The NPs are taken up by macrophages in a corona-dependent manner.^70^ Surface engineering of NPs with the correct chemical motif can be used to create a tunable BC which can prevent the recognition and its interaction with macrophages. An *in vitro* cell uptake study in monocyte/macrophages is a routine experiment that is used as a surrogate to screen NPs for their stealth properties. Low macrophage uptake of NPs *in vitro* under ideal conditions should translate into prolonged circulation in the blood. However, it was reported in a recent study that no correlation was found between the *in vitro* macrophage uptake study of NPs and their *in vivo* circulation time.^71^ Several overlooked experimental factors can result in misleading conclusions. This includes common procedures like the mode of particle administration during *in vitro* studies *e.g.*, in the form of bolus or premixed or bolus mixed *via* aspiration.^72^ The different mode of administration can influence particle-cell interactions and uptake that has been attributed to variation in BC. Therefore, fundamental studies on the bio-nano interactions should be designed with caution.

The BC can have an unanticipated effect by biomimicking exogenous and endogenous substances. Immune-mapping techniques have been used to investigate epitope presentation by NP-adsorbed proteins to cells.^73^ Adsorption of certain endogenous proteins like lipoproteins can inhibit immune activation. NPs which are labeled with certain immune-compatible proteins in the human body are mistaken for endogenous particles and therefore evade the immunological response. A broad range of unanticipated immune reactions can occur due to the exposure of cryptic epitopes of proteins forming the BC.^74,75^ NPs provide an enormous surface area for adsorption and conformational change in biomolecules which depends on the surface chemistry and the nature of the biological fluid. BC also influences the *in vivo* colloidal stability and drug release from NPs.^76^ The size of NPs and adsorption of immunoglobulins can also significantly reduce the vascular adhesion of NPs (90% reduction in case of PLGA NPs).^77^ BC can also influence margination of NPs away from the endothelial wall in the lumen of blood vessels and capillaries resulting in reduced endothelial interaction and extravasation.^78^ Study of the nano-bio interface is therefore critical because it influences the fate of NPs as they move through different compartments of the body with a myriad of biomolecules and biofluids to interact with.

A nearly complete absence of BC can enhance targeting of NPs remarkably and a classic example is the case of core crosslinked polymeric micelles composed of poly(ethylene glycol)-*b*-poly[*N*-(2-hydroxypropyl)methacrylamidelactate] (mPEG-*b-p*(HPMAm-Lacn)) copolymers. They have been successfully used to deliver docetaxel showing dramatic tumour regression in a mice model after a single intravenous injection and leading to 100% survival.^79^ This has been attributed to the enhanced tumour retention of the nanoformulation and its anti-stromal effect. It was well tolerated by healthy rats as compared to the marketed formulation of docetaxel (Taxotrene). A label-free proteomic study shows a negligible amount of adsorbed proteins on the surface of the polymeric micelles which could be a possible reason for their enhanced antitumoral effect.^80^ The micelles in blood plasma were found to retain their nano-size which is an indicator of the colloidal stability of NPs in complex media. In clinical studies, this nanoformulation (CPC634; currently undergoing phase II efficacy trials) exhibited enhanced intra-tumoral drug accumulation and a lower incidence of neutropenia.^81^ A low neutropenia has been associated with a low *C*_max_ of the released drug. This exemplifies the critical role of BC in tumour-targeted NPs.

### 3.2. Role of BC in the immunocompatibility of cancer nanomedicine

Different BCs can activate different immunological pathways depending on the protein adsorbed.^82^ The long circulation behaviour of Apo E pre-adsorbed graphene/gold nanoparticles has been attributed to the lack of complement activation.^83^ Many complement proteins act as opsonins by marking the NPs for rapid clearance from circulation. The complement protein adsorbed on NPs is an important determinant of recognition and clearance by macrophages and therefore plays an important role in the biofate of NPs.^70^ The uptake of superparamagnetic dextran iron oxide (SPIO) was reduced by 95% in C3 deficient mice which demonstrates the role of complement activation in opsonization.^84^ Complement mediated uptake of SPIO was also blocked by EDTA (a complement inhibitor) in blood from healthy volunteers and cancer patients. There is significant variability in complement reactivity towards NPs in the general population which is found to be independent of age and gender.^85–87^ Differential complement activation is a critical determinant of the individual variation in innate immunity. The individual variability in complement and the components of innate immunity can result in variability in the biofate of NPs, including its clearance and pharmacokinetics.

Natural antibodies in the blood adsorb on NPs and can act as an inducer of complement activation. The complement-mediated opsonization of iron oxide (SPIO) NPs by the third complement C3 is dependent on the natural antibody present in the BC.^88^ The natural antibody in plasma was found to be the link between BC and C3 mediated opsonization. Complement activation and internalization *via* opsonization can be triggered by the presence of very few antibodies on the surface of NPs. The activation process of complement by the three pathways (classical, alternative and lectin) converges at a point where C3 is cleaved by the C3 convertases assembly into C3a, C3b and iC3b. The C3b and iC3b fragments bind and prime an activating surface like NPs which aid in cellular biorecognition *via* Fc and complement receptors for opsonization by immune cells.^89^ The C3 adsorption on the surface and C3-mediated biorecognition are key events that dictate the biofate of NPs. Natural antibodies in blood critically influence complement-mediated NP opsonization predominantly by an alternative pathway.^88^ Variation in blood proteome can contribute to individual variability in the cell uptake of NPs. This has been demonstrated by a significant increase in the uptake of NPs at an artificially elevated level of IgG by the cells expressing IgG receptors.^90^ Doubling the IgG concentration resulted in a 40-fold increase in the fraction of antibodies in BC. This significantly increases the cellular uptake of NPs because the adsorbed IgG acts as an opsonin and promotes opsonization *via* Fc receptors present in cells. Individual variability in the adaptive immunity may be one of the factors for the life-threatening infusion reaction observed in some patients treated with PLD.

There are contradictory reports on the effect of surface adsorption of potential opsonins on NPs and their clearance from circulation. It has recently been reported that the complement system has a negligible influence on the circulation time of both PEGylated liposomes and their non-PEGylated counterpart.^91^ Complement activation was unable to explain the pharmacokinetic clearance of liposomes in rodent models. Maintaining circulation stability by controlling the surface properties is critical for the efficient delivery of NPs to the tumour tissue.^92^ Studies on C3^−/−^ knock-out animals show that the complement proteins have no role in the clearance of PEG-coated or uncoated NPs with preadsorbed clusterin (Apo J).^93^ This has been attributed to the non-specific binding of antibodies. Antibodies that bind *via* specific epitopes on NPs are only marked for opsonization.^94^ Screening for background immunity of patients and identification of specific biomarkers which influence the immunological consequence of NPs are therefore required for predictable outcomes in nanomedicine.

## 4. Pharmacokinetic, pharmacodynamic and therapeutic efficacy of nanomedicine

### 4.1. Pharmacokinetic and pharmacodynamic (PK/PD) variability in the use of anticancer nanomedicine

One of the impediments in the clinical success of NPs is the high inter- and intra-variability in their PK/PD. In a PK meta-analysis study, significant inter-patient variability was observed in the covariance coefficient (% CV) of the plasma concentration-time AUC of a liposomal anticancer drug when compared to a lipid-free formulation.^95^ The PK variability has been associated with factors such as the age, gender, body weight, cancer type and monocyte count.^66,96,97^ Significant variability in PK translates into high variability in the efficacy and toxicity of NPs.^95,98,99^ Clinically significant variability in clearance of PLD (15.3-fold) has been observed which was not affected by a dosing schedule based on the body surface area.^66^

The clearance mechanism of PLD is different from that of free drugs. PLD is predominantly cleared by the MPS whereas free drugs are cleared by metabolism in the liver. PEGylation significantly influences clearance. Therefore, PK and PD aspects of PLD are quite different from those of the free drug. The cytotoxic effect of PLD on Kupffer cells, which are resident macrophages in the liver, has been observed in a mice model which results in a longer circulation time of a subsequently injected PEG-liposome formulation.^100^ The change in circulation time was observed between day 3 and 14 after the first dose and subsequently, the effect was lower between days 21 and 28. In a clinical setting, however, this may not be observed due to a 3 week gap between the chemotherapy cycle for Doxil™. The significantly delayed systemic clearance of bacteria also provides indirect evidence for the loss of phagocytic activity of macrophages indicating the toxic effect of PLD on macrophages.^101^ This is a PLD-specific phenomenon and is not attributed to bone marrow suppression (a common side effect of doxorubicin) as it is not observed in the lipid-free drug. An interesting meta-analysis study shows that there is an inverse relationship between the clearance of NPs and inter-patient variability in PK which has important implications in the development of NPs.^102^ While targeted delivery aims to increase the circulation time by reducing the clearance of NPs, however, this may result in interpatient variability in PK and PD (toxicity and response).

The clearance of NPs by cells of the MPS also depends on the natural immunity of an individual. Across multiple species, a strong correlation has been observed between MPS function (both phagocytosis and ROS activity of blood monocytes and dendritic cells) and the clearance of PLD.^103^ Therefore, phenotypic markers for measuring cellular functions can be used to predict the clearance and adjust the dose for personalized medicine. A need for dose adjustment is proposed for patients with liver metastasis where a significant NP clearance is observed due to a high MPS activity.^99^ Therefore, there is a compelling need to adjust the dose based on MPS functions for positive clinical outcomes. This is in stark contrast to the activity of free drugs which shows lower liver metabolism by hepatic enzymes during liver metastasis.^104^ The uptake of PLD by peritoneal macrophages has been reported to induce tolerogenic M2 macrophages and cytokine release by these macrophages can lead to a secondary release of CCL2 (CeC motif chemokine ligand 2 also called monocyte chemotactic protein 1 [MCP-1]). Clinical observations show that in patients with ovarian cancer, plasma clearance of NPs correlates with the plasma CCL2 and monocyte count.^105^ This is substantiated by the fact that an altered clearance of PLD has been observed in CCL2 knock-out mice.^106^ The particulate nature of NPs is responsible for the activation of the immune system which results in substantial variability in PK parameters depending on the patients’ adaptive immunity which is also influenced by the disease state.

The pharmacokinetic characteristic of the carrier dictates the clearance and distribution of the encapsulated drug. It has been demonstrated in a mouse model that PLD can preferentially accumulate in adipose tissue than in muscles. This results in a high volume of distribution and low plasma drug concentration and therefore a reduced efficacy due to poor tumour accumulation.^107^ In human PK studies, a 10-fold difference in plasma exposure between obese patients as compared to normal-weight patients has been observed.^108^ PK variability among obese patients is attributed to faster clearance of NPs by the MPS.^109,110^

Obese patients with cancer show an enhanced level of hormones and chemokines which can influence MPS function.^107^ Patients with high estrone levels show higher MPS activity and lower drug exposure of the encapsulated drug. A high level of hormones and chemokines can modulate MPS function in obesity which is commonly observed in patients with endometrial and ovarian cancer. *In vitro* studies show that estrogen can stimulate the phagocytic activity of the MPS.^111,112^ Individualization of dose depending on the serum hormone concentration and markers of MPS function has also been proposed.^113^ This is based on a strong correlation between serum estrone and PK that has been observed in obese patients (with ovarian and endometrial cancer) who received monotherapy of PEGylated NPs. A high dose of NPs is required in obese patients to achieve plasma exposure equivalent to normal-weight patients. The presence of tumour can influence the local and global immune system that can substantially influence NP clearance. It has been observed that the presence of M2-like macrophages in a tumour model resulted in significant clearance of PRINT hydrogel nanoparticles into the liver and spleen.^114^Table 2 highlights the reasons for PK/PD variability in cancer nanomedicine.

**Table 2:** Summary of the possible reasons for variability in PK/PD of cancer nanomedicines

Reasons for variability in PK/PD	Ref.
Heterogeneity in tumour type influence tumour accumulation by the EPR effect	115
Immune status of the host and the tumour microenvironment (TME)	96, 103 and 114
An inverse relation between liposomal clearance and PK variability has been observed	102
Co-morbid conditions which modulate the MPS system like obesity, diseases which modulate the hormone level, *etc.*	107
Cycle-dependent change in the monocyte count due to the cytolytic effect of the anticancer drug	65
Bodyweight, age and gender modulate the immune system and can influence clearance and distribution	66 and 98
Presence of pre-existing anti-PEG antibodies promotes complement activation-mediated NP clearance	116 and 117
Anti-PEG antibodies and complement-mediated liposomal membrane damage and drug release	56 and 118

### 4.2. Predictors of nanomedicine efficacy and the therapeutic index

The altered distribution of NPs is responsible for their enhanced tumour accumulation. One of the critical questions is whether the accumulation of NPs is a surrogate for the therapeutic efficacy of NPs or are traditional PK parameters a better predictor of efficacy. Both approaches for efficacy assessment have their drawbacks. The assessment of PK parameters as a predictor of efficacy is based on the principle that the plasma concentration is proportional to the target drug concentration following the first-order kinetics. Logically, the same principle cannot be applied for NPs due to an altered pharmacokinetic distribution and enhanced tumor accumulation by the EPR effect. One of the striking observations is that a high plasma concentration doesn't necessarily result in a high tumour drug concentration as expected.

**Fig. 2:**
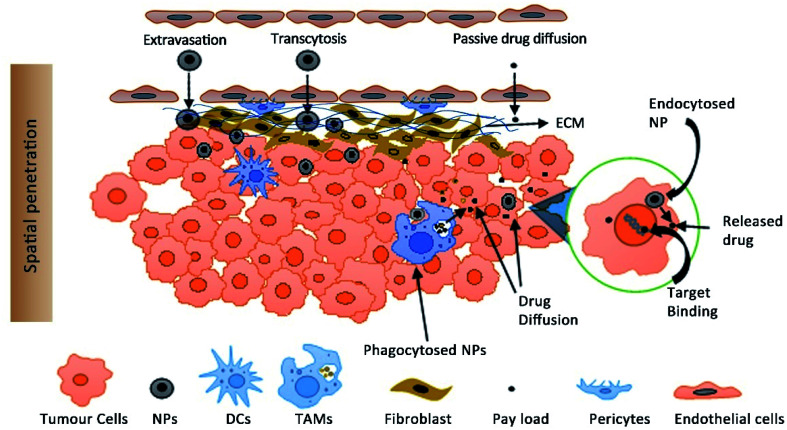
The distribution of NPs from the blood into tissue space includes the process of extravasation, transcytosis and passive diffusion of the free drug (released from NPs). Target binding of the drug includes the process of tumour tissue penetration through the extracellular matrix (ECM) into the interstitial fluid, diffusion and endocytosis, TAM-mediated drug release, intracellular release and target binding (DNA for *e.g.*) of free *vs.* encapsulated drugs. The diagram is not drawn to scale. (TAMs, tumour associated macrophages; NPs nanoparticles; DCs, dendritic cells).

Moreover, it is the free drug that is bioactive and not the encapsulated drug and the determination of plasma drug concentration includes free plus the encapsulated drug. Marketed PEG liposomes show insignificant drug leakage from the liposomes in blood plasma which shows that most of the drug is encapsulated (∼1000 fold higher than free drug in plasma).^119^ It is extremely challenging to determine the free drug concentration at the tumour site which is the most likely predictor of efficacy. The cell uptake of NPs by TAMs present in the TME can also act as a reservoir for the slow diffusion of the encapsulated drug and shows its cytotoxicity on the nearby cancer cells (Fig. 2).^120,121^ This has a direct impact on the efficacy as the intratumoral accumulation of NPs is reduced upon depletion of TAMs.^120^

NPs can also act as a vehicle for intracellular delivery of drugs with poor cell membrane permeability.^122,123^ Laginha *et al.* demonstrated that the amount of free drug doxorubicin from Doxil™ in the cellular nucleus as a fraction of the total free drug in a xenograft model was found to be 40–50%.^124^ The kinetics of encapsulated and free drugs present systemically and intratumorally should be determined for a good PK-PD model.^125^ PK-PD models are used to predict the target drug concentration; however considering the variability at multiple levels of NP distribution both vascularly and intratumorally, validation of these approaches is extremely challenging. NPs which accumulate at the vicinity of the cancer cells should either be taken up by the cell or should release the drug for diffusion and target binding. The effect of the physicochemical properties of NPs and their *in vivo* disposition including tissue distribution and extravasation, tumour tissue penetration, diffusion and endocytosis of free *vs.* encapsulated drugs is required for reliable prediction of efficacy (Fig. 2).

The efficiency of tumour delivery by using a novel PK metric has been proposed which is called the relative distribution over time (RDI-OT).^126^ RDI-OT is defined by the ratio of the tissue drug concentration to plasma concentration at each time point. While standard PK metrics like AUC show that NPs demonstrate efficient tumour exposure, RDI-OT shows free drugs to have better tumour exposure in 8 out of 17 small molecules (SM) than the corresponding NPs tested. The efficiency of the formulation can be evaluated at each time point. High efficiency of SM was observed from 0–6 h in all the tested formulations. However, this PK metric needs to be validated in different tumour models and by correlation with PD like tumour regression and progression-free survival (PFS).

The markers of the therapeutic index in nanomedicine are disputable. The standardization of methods of analysis used to compare therapeutic effectiveness is lacking due to the absence of a regulatory framework by the FDA. In the absence of a regulatory framework, benefit-risk assessment of nanomedicine is based on a case-by-case basis which is time-consuming and requires technical advice from an expert panel. Regulatory bodies still look for the traditional PK parameters to analyze nanomedicine. It is noteworthy that a similar PK profile does not translate into similar extravasation of NPs (which is dependent on the tumor architecture and TME).^127^ However, a high systemic exposure of PLD and a high occurrence of hand-foot syndrome (a side effect of PLD) correlate independently with PFS in clinical studies on patients with advanced breast, endometrial or ovarian cancers.^128^

## 5. Immunological interactions of nanomedicine

### 5.1. Influence of the host immune system and tumor immunology on the fate of NPs

One of the barriers in the safe clinical applications of NPs is the immunological barrier.^129^ The interaction with the immune system can have unanticipated consequences on the fate of NPs. A recent finding suggests that the immune status of the animal model is a critical biological variable that effects cancer-targeting studies of NPs.^130^ The differential retention of NPs across animal models (with different immunological background strains) suggest that the host immune status is a key determinant of the fate of NPs. The interconnection between the host and the local immunity of the TME makes it even more complex.

One of the least known aspects of nanomedicine is its interaction with tumour immunological milieu. The physiological restructuring of the tumour immunological milieu in response to NP deposition can have a positive or negative impact on cancer therapy. On the one hand, it has the potential for NP-mediated cancer immunotherapy and, on the other hand, NP-mediated immunosuppression can have a pro-tumoral effect.

The interaction between NPs and tumor-infiltrating immune cells induces a cytokine milieu in the TME which determines the biofate of nanomedicine.^130^ The tumour immunological milieu is believed to contribute to the suboptimal efficacy of therapeutic NPs. It has been reported that the suppression of antitumour immunity is associated with the pro-tumoral effect of PEGylated liposomal doxorubicin (PLD).^131^ PLD reduces IFN-γ production by TAMs and cytotoxic T lymphocytes which is essential for anti-tumour immunity. The mechanism is based on the uptake of PLD by the TAMs in the tumour microenvironment and its polarization from a tumour suppressive and inflammatory M1 macrophage to an anti-inflammatory M2 phenotype and a global downregulation of inflammatory cytokine secretion by T cells.

The notion that active targeting is due to the binding of antibodies present on antibody-labeled NPs with the antigens overexpressed on tumour cells has been challenged by a recent report. A significant accumulation of antibody-labelled NPs (in comparison to unlabeled NPs) in the tumour tissue was found to be dependent on the immune cells in the tumour milieu and not on the antigen–antibody interaction observed in *in vitro* studies.^130^ It was also reported recently that active targeting of NPs shows extremely low targeting properties as very few NPs (14 of 1 million NPs) interacted with cancer cells.^23^ This exemplifies a significant challenge in the delivery of NPs to cancer tissues.

The interaction of NPs with the immune system also presents huge possibilities for immunotherapy. It has been observed that irrespective of the tumor retention potential, NPs (both plain and antibody-labeled) can induce a similar immune response, T cell infiltration and tumour inhibition.^130^ An anti-tumor immune response mediated by CD8+ T cells can be induced by NPs without the requirement of any bioactive payload. NPs have the potential to modulate both systemic and local immune effects on tumour growth inhibition which may have potential for cancer immunotherapy.

The TME has an immunosuppressive environment characterized by a hypoxia-induced cytokine cocktail which negatively regulates tumor antigen presentation.^132^ The hypoxic environment also restricts the CTL count; however, its cytolytic capacity is not compromised.^133^ The host immune status and the tumor immunological milieu are critical variables for NP-based cancer therapy. TAMs, MDSC and Treg cells are the key effector cells responsible for an immunosuppressive TME which are the possible target cells for immunomodulation.^134,135^

### 5.2. Immunological consequence of NPs may promote the pro-tumoral effect

NP-mediated immunological mechanisms that inhibit antitumoral immunity have been demonstrated to cause the pro-tumoral effect of NPs. The polarization of TAMs from an anti-tumoral M1 phenotype to a pro-tumoral M2 phenotype by PEGylated liposomes has been reported.^136^ PEGylated liposomes have shown a pro-tumoral effect in an immunocompetent mice model, subcutaneously implanted with TC-1 cells.^131^ The pro-tumoral effect is attributed to the suppression of Th1 cytokine (IFN gamma) and low CTLs in the tumour tissue as compared to vehicle control. It was also found that the pro-tumoral effect depends on the type of implanted tumour and not on the background immunity of the selected animal model. Liposomes not only increase the primary tumour, they also increase the peritoneal metastasis of orthotopic implanted cells (ID8-VEGF-GFP) in a mice model.^136^ Complement activation has also been implicated in the pro-tumoral effect of NPs. Tumour accumulation of empty poloxamine 908 coated polystyrene NPs and activation of intratumoral complement against long circulatory NPs is thought to be responsible for the pro-tumoral effect.^137^ The complement protein C5a induces an immunosuppressive environment by the recruitment of MDSCs and suppression of CD8+ T cells.^138^ Activated components of complement can influence various stages of carcinogenesis by evading immune recognition, promoting angiogenesis, cell migration, and activating growth factors and preventing apoptosis.^139^ The pro-tumoral effect of NPs is a matter of great concern for the translation of NPs, which could partially explain the lack of efficacy. In the above examples of the pro-tumoral effect of NPs, studies were performed with placebo NPs without the drug. The presence of drug may exert a cytotoxic effect on the immune cells responsible for a tumour suppressive environment which may negate the influence of an immunosuppressive state and therefore further studies are warranted to fully establish the pro-tumoral consequence of NPs.

## 6. Relevance of preclinical models in cancer nanomedicine

### 6.1. Pre-clinical models to predict the targetability and efficacy of cancer nanomedicine

NPs show strong interactions with the host immune system. The interconnection between the host and the local immunity of the TME makes it even more complex. Questions have been raised against the relevance of preclinical models in cancer nanomedicine. Pre-clinical studies of NPs are often performed on patient-derived xenograft models for which immunocompromised animals are used due to a conducive immunosuppressive environment for cross-tissue grafting. The interaction of NPs with the host immune system has a bearing on the fate of NPs which challenges the basic premise of tumour targeting studies in immunocompromised models. The tumour growth and progression in the absence of immune pressure lacks the complexity of a clinical tumour. The process of immunoediting which happens in clinical tumours might not happen in the preclinical models. The ratio of tumour to body mass also varies significantly between humans and rodent models. In mouse models, the tumour volume may grow to a size of about 10% of the body mass.^38^ This can significantly alter the body distribution and increase tumour accumulation of NPs, accentuating the EPR effect. Human tumours can vary in size, but those eligible for nanodrug delivery usually have a tumour weight of not more than a few grams with a low tumour to bodyweight ratio. Preclinical studies are more focused on treatment outcomes and less on the host immune system and its interaction with NPs. The preclinical testing of NPs is usually performed in comparison to a drug monotherapy as a control that is clinically irrelevant. Current standards of treatment are generally combination therapies and therefore, preclinical studies add very little value to clinical progress and translation.^15^

Recently, it has been observed that the mouse model with an intact immune system shows high tumour retention of NPs as compared to immunocompromised models.^130^ Tumour-associated phagocytic cells also have a significant influence on the tumour retention of NPs. Species differences in complement-mediated recognition and opsonization of SPIO NPs by leukocytes have been observed between humans and mice.^84^ The number of immune cells especially neutrophils is significantly increased in tumour-bearing mice which results in enhanced uptake. The use of immunocompromised mice models for cancer also prevents the evaluation of the immunosuppression-induced pro-tumoral effect of PEGylated NPs. The pro-tumoral and immunosuppressive nature of the PEGylated NPs was never the focus of preclinical studies and these factors were overlooked possibly resulting in overhyped conclusions which could not be replicated in clinical trials. Further, the dose used in preclinical studies is typically much larger than the clinical dose, which might be the reason for the cytotoxic effect of NPs overriding their immunosuppressive effect.^140^ The dosing interval between chemotherapy is usually 2 to 4 weeks (recovery phase),^141^ which in the case of mouse models is as low as three days. The dose and dosing interval can influence the monocyte count and antibody generation that can affect the clearance of NPs and the ABC phenomenon.^61,105^ The extended dosing interval on the other hand can influence the recovery of cancer cells that may also gain drug resistance.^142^ So, an aggressive dosing schedule in a mouse model can influence efficacy. The blood proteome and hemorheology of rodents differ from humans which can also influence the BC-mediated biological interaction of NPs.^143,144^

NP disposition is a function of its physicochemical characteristics and the global immune status of the host (Table 3). The presence of tumour can have a dramatic influence on particle clearance due to a change in the local and global immune system in preclinical models.^145^ The tumour burden can polarize the immune system to a Th2 phenotype which can influence the biofate of NPs. The short plasma circulation and enhanced clearance of NPs by the MPS in tumour-bearing animals were reported to be due to the polarization of macrophages to a M2 phenotype. This shift in the immune response which prevents a cell-mediated response in the TME has also been reported in humans.^146^ Even among immunocompetent mice, the global immune status of the mouse strain can vary considerably.^147^ A mouse strain with a predominantly Th1 response (*e.g.* C57BL6) shows a significantly slower rate of clearance than a Th2-prone mouse (*e.g.* BALB/c).^148^ This has been attributed to the polarization of macrophages to a M1 phenotype by Th1 cytokines and the tolerant M2 phenotype by Th2 cytokines.^149^ However, the particle clearance by a Th1 biased strain is similar to that by a Th2 strain following tumour induction which exhibits a shift in the immune response with cancer progression that can dramatically influence particle clearance.^145^ A deeper understanding of the molecular aspects of this shift in immune response will help optimize nanodrug delivery. The pivotal role of immune system in the kinetics of NPs and tumour immunobiology is a critical aspect that has often been overlooked in preclinical studies.

**Table 3:** Animal models and their immunological effect on NPs^a^

Animal model	Immune competence	Tested NP type	Remarks	Ref.
CARPA model	PIMs are resident macrophages present in pigs and not in humans and rodents	Doxil™	NP clearance by PIMs is responsible for the infusion reaction. A model to assess the risk of HSRs	48
C57BL6 mice	Th1-dominant strains	PEGylated 300 nm hydrogel NPs	M1 macrophage polarization by Th1 cytokines results in a low particle uptake	148
BALB/c mice	Th2-dominant strains	PEGylated 300 nm hydrogel NPs	M2 macrophage polarization by Th2 cytokines results in a higher nanoparticle uptake	148
BALB/c nu/nu mice	T cell-deficient	PEG-liposomes	ABC phenomenon observed by a T cell-independent B cell response	151
SCID mice	(T and B cell-deficient)	PEG-liposomes	ABC phenomenon is not observed and B cells are a prerequisite for ABC	151
Foxn1nu (athymic nude, C57BL/6J background) mice	T cell-deficient	PEGylated print hydrogel particles	A shift from Th1 to Th2 immune response was observed during tumour progression	145
FVB/N	Immunocompetent mice	Antibody-labelled modified magnetic NPs	Retention of NPs in the TME is dependent on multiple lineages of immune cells	130
Athymic nude mice	Lack mature T cells	Antibody labelled modified magnetic NPs	Immune-mediated tumour suppression by NPs was not observed because of the lack of an intact immune system	130
FVB/N	Immune competent mice	Antibody labelled modified magnetic NPs	Pharmacological inhibition of the host immune system reduces the tumour retention of NPs	130
Wistar rat	Immune competent rat	PLDs	Mouse models show a very low complement level and rat models are more relevant	56 and 150

a Pulmonary intravascular macrophages (PIMs), complement activation-related pseudoallergy (CARPA), hypersensitivity reaction (HSR), accelerated blood clearance (ABC); NP nanoparticle; enhanced permeability and retention (EPR); tumour microenvironment (TME); particle replication in nonwetting templates (PRINT).

Species-specific differences in complement activation affects complement-mediated liposomal membrane damage and the release of drug from PLDs.^56^ Due to the low cytolytic activity of anti-PEG antibodies in mice, they were not found to be suitable models to study complement-induced liposomal lysis. This suggests the role of interspecies difference in human translational studies. Wistar rats were found to be efficient in demonstrating the complement-mediated change in pharmacokinetics and toxicity of PLDs. The complement level of common mice strains is very low relative to humans and therefore complement related studies in mice models may not be physiologically relevant.^150^

## 7. Future outlook

### 7.1. Rationale for the design of a fixed molar drug ratio in NPs: case study of Vyxeos (liposomal combination of cytarabine and daunorubicin)

**Fig. 3:**
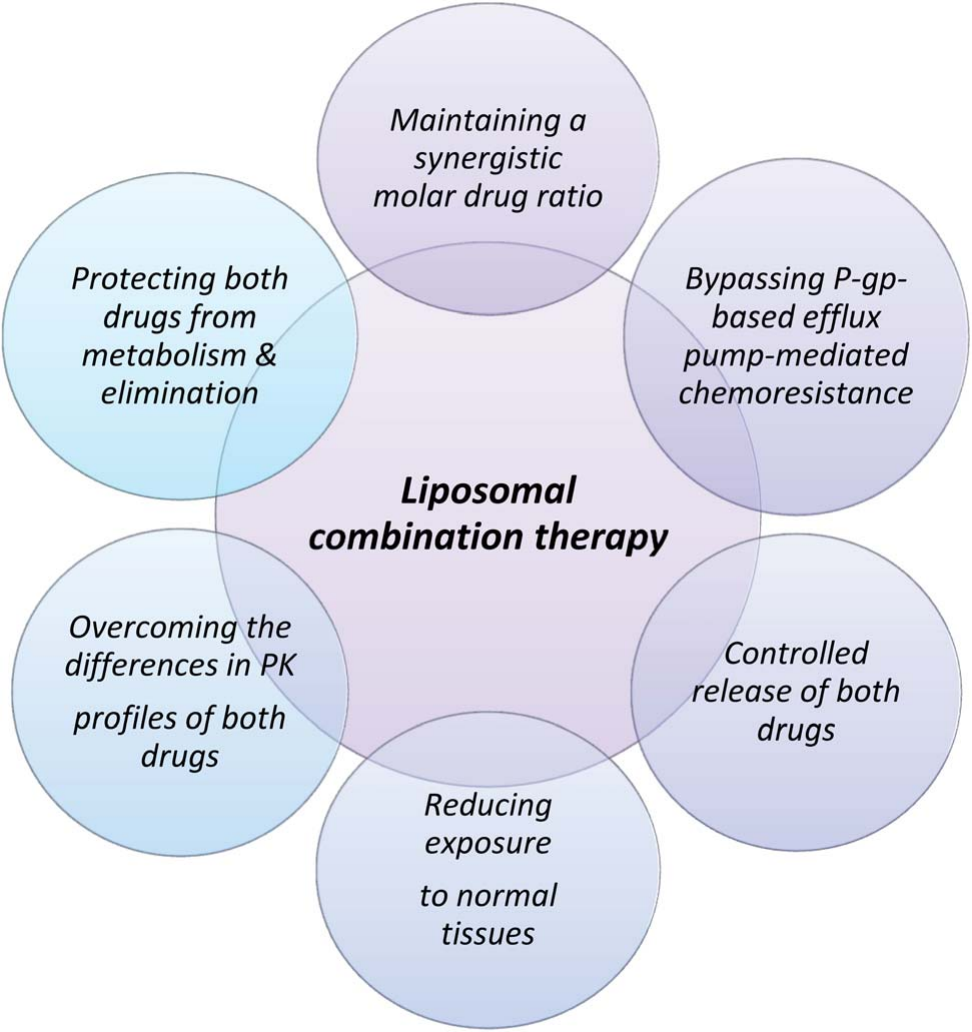
Proposed rational for the design of fixed molar drug ratios for therapy using Vyxeos (liposomal combination of cytarabine and daunorubicin).

The theoretical basis for the use of a combined drug regimen in cancer chemotherapy is to improve efficacy, reduce drug resistance and decrease toxicity by reducing the dose. The conventional standard of care treatment of acute myeloid leukemia (AML), a heterogeneous cancer is by a 7 + 3 regimen. This is optimized combination therapy with two drugs developed by a trial-and-error method.^152^ The 7 + 3 regimen involves 7 days of continuous infusion of cytarabine in combination with 3 days of concurrent intermittent dosing of daunorubicin.^153^ Ratiometric delivery of drugs is based on the rationale that a combination of molar ratios of drugs can have synergistic action relative to using individual drugs at the maximum tolerable dose.^154^ Vyxeos was developed with the idea that a fixed molar drug ratio of 5 : 1 packed in liposomal vesicles would provide better efficacy and tolerability to the drug administered historically by the standard 7 + 3 regimen. The proposed advantage of using a liposomal fixed molar ratio of the drug is highlighted in Fig. 3. This ratio was found to maximize efficacy and reduce antagonism during cytotoxic study in a panel of cell lines.^155^ Cytarabine is loaded passively in liposomes whereas daunorubicin is loaded actively using copper gluconate as a buffer to improve drug retention in liposomes by metal complexation with copper.^155^ The synergistic effect of the ratiometric delivery has been observed in a preclinical model when compared to separate doses of liposomal cytarabine and daunorubicin that showed a significantly lower antitumoral effect than Vyxeos.^155^ In the phase III studies, clinically significant overall survival was observed in comparison to the standard 7 + 3 regimen (CLTR0310-301). Based on the relative molar ratio of the drugs, the action may vary from synergistic to antagonistic effects. Although the molar drug ratio of 5 : 1 shows maximum synergistic activity *in vitro* it is difficult to maintain *in vivo* ratiometry by traditional chemotherapy using iv infusion due to the difference in the pharmacokinetics of the two drugs which may eventually lead to an antagonistic dose ratio.^154^ Liposomes at a lipid ratio of 7 : 2 : 1 of distearoylphosphatidylcholine (DSPC), distearylphosphatidylglycerol (DSPG), and cholesterol provide the requisite biophysical properties for homing the drug in the required 5 : 1 ratio to maintain the synergistic ratio for an extended duration that has been used to resolve the issue of delivering the synergistic combination to the target cells.^156^ It is a non-PEGylated liposome that remains in a gel state at body temperature and provides the required *in vivo* stability.^157^ The most important attribute of using a liposomal carrier is to deliver the required synergistic ratio, which brings predictability and minimizes antagonism between the two drugs which is difficult with conventional chemotherapy.

It has been observed that 24 hours post-administration the molar ratio of the drugs was maintained between 5 : 1 to 9 : 1 (in the synergistic range) whereas, within 15 minutes of administration, the ratio of the free drug was changed substantially from the synergistic ratio.^155^ It has also been claimed that liposomes can accumulate preferentially in the malignant myeloblast cells and have demonstrated significant cytotoxicity on leukemia precursors than normal hemopoietic precursors.^158^ However, it has been observed in preclinical studies that liposomal formulation shows a lower nadir of all blood and precursor cells as compared to the 7 + 3 regimen.^159^ Clinical data also reveal the fact that there is a delayed recovery of platelets and neutrophils following a prolonged period of thrombocytopenia and neutropenia on the administration of liposomal formulation.^160^

### 7.2. Personalized drug therapy using NPs

**Fig. 4:**
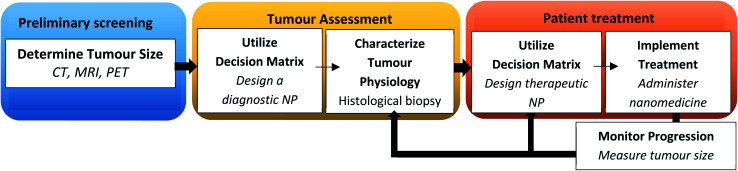
Flow diagram illustrating a decision matrix for personalizing nanotherapeutics in a clinical setting. Adapted with permission from ref. 163 Copyright (2016) National Academy of Science of the USA.

The traditional method of designing NPs of defined physicochemical properties as a ‘one-size-fits-all’ approach for all cancer types has yielded limited success. A disease driven-approach has been proposed for the rational design of NPs as against a formulation-driven approach used traditionally.^161^ This strategy relies on the pathological changes associated with the disease to design NPs with desired characteristics.^162^ A change in pathophysiology at different stages of tumour progression can influence size-dependent particle retention and penetration of NPs.^163^ It has been reported that gold NPs with a large particle size is more suited for accumulation in a large tumour volume, but this occurs at the expense of NP penetration in tumour tissues. The smaller size of NPs is suited for low tumour volumes with greater depth of penetration but may carry a low payload compared to larger particles. Therefore, there is a trade-off between the intended function and optimal drug delivery by NPs. A decision matrix for personalized therapy based on the tumour volume, particle characteristics, uptake rate, accumulation and permeation is shown in Fig. 4.^163^

This involves image-guided techniques, biomarkers and biopsies to characterize various barriers such as the permeability characteristics of the endothelium, barrier function of the tumour extracellular matrix, interaction of NPs with the host immune system and the tumour immunological milieu. This may aid in predicting the treatment response and variability in the EPR effect based on which patient population can be stratified with a greater likelihood of success in clinical trials.^164^ Imaging techniques such as positron emission tomography (PET) and high-resolution confocal microscopy have been used to quantify tumour-associated macrophages (TAMs) in a preclinical model which correlates with particle deposition at the tumour site.^165^ The increased cellular density of TAMs has been associated with the EPR effect. Magnetic resonance imaging has also been used to track the EPR effect with a high degree of predictability.^164^ Similarly, ferumoxytol, a clinically approved 30 nm magnetic nanoparticle has been used to predict the tumour response by the EPR effect.^121^

Characterizing the biomarkers of immunological interactions and biophysical aspects of EPR can be used to guide the design of NPs for personalized therapy. Immunological and imaging tools can assist nanomedicine research in patient stratification for tailormade bio-guided solutions for improved efficacy. Optimizing the size and surface properties of NPs depending on the cellular and structural properties associated with the pathophysiological stage of tumour growth has shown to increase tumour targeting by 50%.^163^ The dynamic nature of the dense fibrillar collagen in the stromal compartment which includes the ECM and the stromal cells (consisting of pericytes, (myo) fibroblast cells, endothelial cell, immune cells like the dendritic cells and TAMs) offer different degrees of resistance to the penetration of NPs which varies among tumour types and the stage of the tissue growth.^166,167^ The structural features of the tumour tissue may also change during multiple rounds of chemotherapy. The relationship between the characteristics of NPs with their transport across vascular, extravascular, extracellular and cellular barriers should guide the next generation of NPs with the goal of personalized medicine.

### 7.3. Nano-enabled immunotherapy strategies for targeting the tumour micro environment (TME)

The interaction of NPs with the immune system also presents huge possibilities for immunotherapy. NPs have the potential to modulate the global and local immune effect on tumour growth inhibition that may have potential in cancer immunotherapy. The TME has an immunosuppressive environment characterized by a hypoxia-induced cytokine cocktail that negatively regulates tumor antigen presentation.^132^ The hypoxic environment also restricts the CTL count.^133^ The immune status in the TME supports the growth and proliferation of cells and the interaction with NPs can re-edit the immunological milieu causing a transient immune recognition of the tumor cells by the immune system. Re-educating the immune system against cancer cells is the most natural way to treat cancer and nano-enabled strategies are explored due to its immunostimulatory properties. A combination of immunotherapy and chemotherapy has also been used by a nano-enabled approach against highly refractive pancreatic ductal adenocarcinoma (PDAC) using synergistic dual drug delivery.^168^ Oxaliplatin was used to induce immunogenic cell death (ICD) whereas another drug indoximod was used to block a regionally overexpressed immunosuppressive enzyme, indoleamine 2,3-dioxygenase 1 (IDO1) (which interfere with cytotoxic T cells (Tc) development and induce immunosuppressive T regulatory cells (Treg)). ICD is characterized by the expression of calreticulin during oxaliplatin-induced apoptotic cell death which acts as an ‘eat me’ signal for dendritic cell uptake. On similar lines, a dual drug delivery using drug conjugated polymeric micelles of doxorubicin (ICD inducer) and indoximob shows a synergistic effect causing significant regression of the tumour burden in a rodent breast cancer model. Doxorubicin induces ICD-triggered infiltration of CD8+ cells and secretion of IFNγ while indoximob inhibits the Treg cells.^169^ Nano-enabled re-programming of the tumour immunological environment for anticancer immunotherapy and drug delivery is currently being investigated aggressively and has been reviewed elsewhere.^170,171^ A promising nano-enabled immunotherapy strategy to polarize and modulate the pro-tumoral M2-phenotype into anti-tumor M1-like tumour associated macrophages (TAMs) in the TME is highlighted in Table 4. These observations have opened up a fascinating new area in the field of cancer nanomedicine. The mechanistic aspects of the interaction between the host immune system and the tumour immunological milieu on the biofate of tumour targeted NPs is still vague. Cancer immunotherapy has the potential to radically change the landscape of cancer therapy.

**Table 4:** Some promising nano-enabled strategies for modulating and reprogramming the pro-tumoral M2-phenotype into anti-tumor M1-like macrophages for immunotherapy^a^

Immunogenic payload/delivery to TAMs	Ligand/delivery vehicle	Tumour model/cells	Ref.
Delivering TLR agonist CpG-ODN	Human ferritin heavy chain (rHF) nanocage surface modified with M2pep	4T1 tumor-bearing mice	172
Delivery of dual agonist of the TLR7/8 (resiquimod)	mUNO peptide-guided lignin nanoparticles	CD206-positive M2-like TAMs	173
Specific depletion of TAMs by targeted delivery of dasatinib (competitive inhibitor of the SRC family and ABI tyrosine kinase)	Mannosylated mixed micelles	4 T1 allograft tumor Balb/c mice model	174
Delivery of TLR7 agonist imiquimod	Fe_3_O_4_ polymeric NPs coated with (LPS)- treated macrophage membrane	Orthotopic breast cancer models with 4 T1 cells	175
Inhibitor of CSF1R- and SHP2-present on macrophages	PEGylated phospholipid self-assembly conjugated with CD206 antibody fragments	4T1 breast cancer mouse model	176
Delivery of baicalin (an immunostimulatory flavon), antigenic peptide (Hgp 10025-33, Hgp) and a TLR-9 agonist (CpG)	PLGA nanoparticles coated with a galactose-inserted erythrocyte membrane	Murine B16 melanoma cancer model	177
Delivery of IL-12	pH-sensitive poly(RGD-*co*-beta-amino ester)s NPs	B16-F10 cell xenografted tumor mice model	178
Delivery of ibrutinib, an irreversible BTK inhibitor	Sialic acid–stearic acid conjugate modified phospholipid nanocomplexes	S180 tumor-bearing mice	179
Iron chelation induced M1 polarization. PDT induced TAA release and its presentation by M1-like macrophages to stimulate T cell immunity	Iron chelated melanin-like nanoparticles	Orthotopic breast cancer models with 4 T1 cells	180
Delivery of 3-methyladenine (a P13K γ small molecule inhibitor)	Mannose modified porous hollow iron oxide nanoparticles	MDA-MB-231 cells tumor mice model	181

a Murine M2 macrophage-targeting peptide (M2pep); poly(d,l-lactide-*co*-glycolide) (PLGA); stimulating factor 1 receptor (CSF1-R); Src homology region 2 (SH2) domain-phosphatases SHP-1 and SHP-2; toll-like receptor (TLR); Bruton's tyrosine kinase (BTK); photothermal therapy (PDT); tumor-associated antigens (TAAs).

## 8. Conclusion

We have discussed the key outstanding issues in different areas of contemporary relevance in cancer nanomedicine. Identifying the possible causes of the lack of efficacy is critical as we move forward in our efforts to translate safe and effective nanomedicine. Going back to basics and focusing on the fundamental nano-bio interactions is required to address these issues and to look beyond proof-of-concept studies of NPs.^182^

The fundamental interaction at the nanobio interface is the starting point in the understanding of the biological consequences of NPs. Interactions occurring under static *in vitro* conditions often vary from dynamic physiological conditions.^76^ Interference of NPs in *in vitro* cell-based assays and a lack of standardization of protocols often result in false-positive and contradictory results, making it difficult to predict the *in vivo* consequences of NPs.^183^ Fundamental interactions at the nano-bio interface and their influence on the biofate and immunological consequence of NPs are critical for the immunotherapy-based applications of NPs. The interaction of the host immune system and tumour immunological milieu has an impact on all aspects of targeting including long circulation, clearance, extravasation, infusion reaction, tumour immunosuppression/activation and pro-tumoral effect. The mechanistic understanding of the outstanding issues in cancer nanomedicine has radically changed in the last decade which is highlighted in Table 5. The nano-bio interaction and the complex interdependency of the host and the tumour immune response is by far the least understood of all the problems in cancer nanomedicine. Understanding the mechanism of nano-enabled immunotherapy is currently the most promising avenue in cancer research. Future research on the nano-bio interactions and the immunological consequences of NPs at the fundamental level will be critical to reflect on the most pressing questions in cancer nanomedicine.

**Table 5:** A list of outstanding issues in nanomedicine and our changing perspectives on their mechanistic understanding^a^

Issues in nanomedicine	Previous understanding	Current understanding	Remedial approaches
Biofate of nanoparticles	It is a function of physicochemical properties such as the size, shape, charge, morphology, surface chemistry, hydrophobicity *etc.* of NPs	Type of BC dictates the downstream effects of NPs. The BC fingerprint is determined by the properties of NPs^184,185^	Engineering the surface chemistry for predictive protein adsorption^184^
Stealth	Long circulation was thought to be due to non-fouling surface	Adsorption of dysopsonin *e.g.* clusterin is responsible for stealth^29^	Surface modification for adsorption of biocompatible proteins like dysopsonins^93,168^
Biocompatibility	PEGylation was thought to be non-immunogenic and bioinert	PEG can generate anti-PEG antibodies, cause infusion reaction, and complement activation and ABC^47,54^	Screening of anti-PEG antibodies before using PEGylated carriers^34^
Mechanism of extravasation	Passive targeting due to leaky vasculature	Tumour uptake happens by transcytosis^12^	Transcytosis pathway can be utilized for targeting^186^
NP clearance	NPs are mainly taken up by the cells of the liver and spleen	A large fraction of NPs is taken up by TAMs in the TME	Particles taken up by TAMs can act as a depot for release of the drug^120,121^
Pro-tumoral effect of PEGylated liposomes	PEGylated NPs are safe and biocompatible	Pro-tumoral effect of NPs may occur due to M2 polarization of TAMs but may be tumour specific^131^	NPs which can cause M1 polarization of TAMs can be used for immunotherapy^140^
Altered toxicity profile	Side effects are reduced due to encapsulation and tumour specific accumulation as compared to the free drug	NPs can generate IR and complement activation (CARPA) and skin accumulation causing dermal toxicity not found in the free drug^48,52^	Identifying biomarkers for IR is required. Methods to enhance the EPR effect will reduce skin accumulation^47^
EPR effect	Thought to be the universal gateway for tumour targeting. Was found to be effective in preclinical models	No clinical improvement in efficacy. Inter and intra-tumoral heterogeneity in the EPR effect^38,120^	Stratification of patients based on diagnostic imaging and biomarkers^163^

a TAM, tumour associated macrophage; NP nanoparticle; EPR, enhanced permeability and retention; IR, infusion reaction; BC, biomolecular corona; TME tumour microenvironment; ECM extracellular matrix; CARPA, complement activation-related pseudoallergy; ABC accelerated blood clearance.

## Abbreviations

DDS: Drug delivery systems
BC: Biomolecular corona
TAM: Tumour associated macrophage
NP: Nanoparticle
DC: Dendritic cell
FC: Fibroblast cell
IFP: Interstitial fluid pressure
MDSC: Myeloid-derived suppressor cells
TME: Tumour microenvironment
ECM: Extracellular matrix
NP: Nanoparticle
EPR: Enhance permeability and retention
PEG: Polyethylene glycol
IR: Infusion reaction
PIMs: Pulmonary intravascular macrophages
CARPA: Complement activation-related pseudoallergy
ICD: Immunogenic cell death
ABC: Accelerated blood clearance
PLD: PEGylated liposomal doxorubicin
CTL: Cytotoxic T lymphocytes
Treg: T regulatory cells
MPS: Mononuclear phagocyte system
AUC: Area under the curve

## Funding

This research did not receive any specific grant from funding agencies in the public, commercial, or not-for-profit sectors.

## Conflicts of interest

Authors declare that they have no conflict of interest or financial conflicts to disclose.

## Supplementary Material
